# Scaling and functional morphology in strigiform hind limbs

**DOI:** 10.1038/srep44920

**Published:** 2017-03-22

**Authors:** Meena A. Madan, Emily J. Rayfield, Jen A. Bright

**Affiliations:** 1 School of Earth Sciences, University of Bristol, Bristol, United Kingdom; 2 School of Geosciences, University of South Florida, Tampa, Florida, USA; 3 Center for Virtualization and Applied Spatial Technologies, University of South Florida, Tampa, Florida, USA

## Abstract

Strigiformes are an order of raptorial birds consisting exclusively of owls: the Tytonidae (barn owls) and the Strigidae (true owls), united by a suite of adaptations aiding a keen predatory lifestyle, including robust hind limb elements modified for grip strength. To assess variation in hind limb morphology, we analysed how the dimensions of the major hind limb elements in subfossil and modern species scaled with body mass. Comparing hind limb element length, midshaft width, and robusticity index (RI: ratio of midshaft width to maximum length) to body mass revealed that femoral and tibiotarsal width scale with isometry, whilst length scales with negative allometry, and close to elastic similarity in the tibiotarsus. In contrast, tarsometatarsus width shows strong positive allometry with body mass, whilst length shows strong negative allometry. Furthermore, the tarsometatarsi RI scales allometrically to mass^0.028^, whilst a weak relationship exists in femora (mass^0.004^) and tibiotarsi (mass^0.004^). Our results suggest that tarsometatarsi play a more substantial functional role than tibiotarsi and femora. Given the scaling relationship between tarsometatarsal width and robusticity to body mass, it may be possible to infer the body mass of prehistoric owls by analysing tarsometatarsi, an element that is frequently preserved in the fossil record of owls.

Owls comprise a diverse order of raptors consisting of nearly 250 species with a worldwide distribution and an extensive range of morphologies that make them well suited to their various habitats^1^,^2^,^3^. Characterized by primarily nocturnal or crepuscular activity, owls have evolved keen sensory adaptations that make them formidable night time predators; the ecological counterpart to diurnal birds of prey^4^,^5^,^6^. The phylogenetic position of the order Strigiformes was a topic of great debate among taxonomists for the last two centuries^7^, however DNA and mitochondrial evidence confirms their monophyletic status and phylogenetic position among the other avian orders^8^,^9^,^10^. Owls form a morphologically consistent group whose osteology is easy to distinguish, with characteristics such as large, round forward-facing immobile eyes, more than twice the average size for other birds of a similar size and weight, that sit inside sclerotic tubes fused to the skull and allow them binocular vision; extreme flexibility in the craniocervical region, with the capacity to swivel their heads about their necks up to 270 degrees in either direction—a result of ocular immobility; ears that are frequently placed asymmetrically on either side of the head; and large talons with low curvature relative to other raptors^1^,^4^,^7^,^11^,^12^.

Strigiformes are subdivided into two families: the Tytonidae, comprising 27 species including the barn, grass, and bay owls, and the Strigidae, or true owls, comprising 223 species^4^. The Tytonidae are characterized by large heads with a heart-shaped facial disc, round ear openings, and generally have long legs and powerful feet; barn owls may also be distinguished from true owls by their sternum, which has a broad carina that becomes slightly narrower ventrally, and the ventral edge of which has slight emarginations on either side^4^. The Strigidae, conversely, are characterized by large, round crania with more variable outer ear shape, strong, hooked bills, a round facial disc, a compact body shape, and strong feet with sharp talons; the true owl sternum is narrower dorsally and has two deep emarginations on either side of its ventral edge^4^. Tytonids are medium-sized owls whose habitats range from dense forests to deserts^7^, however Strigiformes are found in nearly all terrestrial habitats ranging from the tropics to the Arctic and as such display a great deal of variation across individual genera from large 3,000 g Eagle Owls to 170 g Little Owls, with substantial differences in diet, ecology, and behavior^7^,^13^.

Hind limbs perform a crucial role in strigiform behavior, particularly prey capture. When hunting, Strigiformes ambush their prey close to the ground and dispatch prey primarily by employing their feet (though occasionally once subdued in the talons, owls will hold prey in their beak and perform a twist with their dexterous necks at the base of the neck of the prey item, possibly in an attempt to break it ref. 11). Strigiform feet have four toes, all of which have distinctly curved, nearly uniform raptorial claws used to strike and grip prey items^1^,^4^,^11^,^14^. In the Tytonidae, digit II and digit III are approximately equal in length with serrations on the underside of the talon on the central toe, but in the Strigidae, digit II is distinctly shorter than digit III^4^. Strigiformes have a digit II claw that is larger than digit III and can be distinguished as birds of prey from this character alone^11^,^15^,^16^,^17^,^18^; they have shorter, more robust toes relative to other raptors, particularly on digits III and IV, in addition to a slightly lower inner claw curvature; they can also rotate digit IV in order for digits II and III to oppose digit I and digit IV, creating a functionally zygodactyl foot^11^,^12^.

The hind limbs of birds must support their body mass and withstand the forces associated with locomotion and hunting. As body mass increases, the strength of support structures must therefore increase correspondingly. Geometric similarity, or isometric scaling, occurs when proportionate dimensions are preserved as size changes. Isometrically, areas, such as the cross-sectional area of the hind limb, scale to length squared, whereas body mass scales to length cubed. Non-isometric, or allometric, scaling occurs when certain proportions change in a regular fashion. In a log-log plot of mass *x* against length *y* where *y* = *bx*^*a*^, isometry is found when the scaling exponent *a* = 0.33. An exponent greater than 0.33 represents positive allometry, where a linear morphological variable increases at a faster rate than body mass. An exponent less than 0.33 represents negative allometry, where a linear morphological variable decreases relative to an increase in body mass. For long columns such as hind limb elements, whose critical morphological variables are length and diameter, a scaling exponent of 0.25 indicates elastic similarity, where structures maintain a constant value of elastic deflection with changing body mass^19^,^20^. In the case of a ratio between two measurements of the limb, such as the ratio of width to length, isometry is represented by a scaling exponent of *a* = 0, with positive allometry represented at *a* > 0 and negative allometry at *a* < 0 (refs 19 and 20).

Recent studies have found that total avian limb length scales with positive allometry^21^,^22^. Studies of the individual bones comprising the limb have shown varying results across the different elements and across phylogenetic groups and locomotor categories. In general, the lengths of the tarsometatarsus and in some cases the tibiotarsus scale with positive allometry, whereas femur lengths scale close to isometry^23^,^24^. There is some evidence to suggest that the lengths of the horizontal bones of the hind limb, the femur and phalanges, scale closer to elastic similarity (*a* = 0.25) (refs 23,24).

The aim of this study is to focus on a single clade and quantify hind limb scaling across a range of owl species with varying adult body mass. Our null hypothesis states that as owls increase in body mass, the length of their hind limb bones (femur, tibiotarsus, and tarsometatarsus) and the robusticity of these bones (ratio of width to length) will scale in order to maintain isometry.

## Results

**Figure 1:**
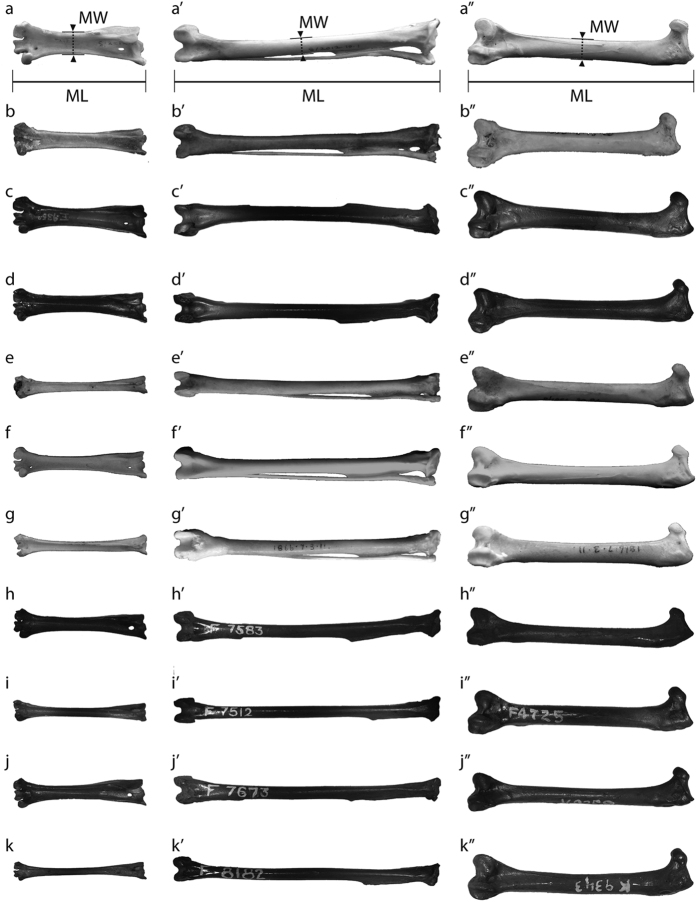
Exemplar right owl limb elements in posterior view, organised by decreasing body mass. Elements are scaled to equal length to highlight differences in robusticity. x = tarsometatarsus; x’ = tibiotarsus; x” = femur. (**a**–**a”**) *Nyctea scandiaca*; (**b**–**b”**) *Ketupa ketupu*; (**c**–**c”**) *Bubo virginianus*; d-d”) *Oraristrix brea*; (**e**–**e”**) *Tyto novaehollandiae*; (**f**–**f”**) *Strix aluco*; (**g**–**g”**) *Tyto capensis*; (**h**–**h”**) *Asio flammeus*; (**i**–**i”**) *Tyto alba*; (**j**–**j”**) *Asio otus*; (**k**–**k”**) *Athene cunicularia. Bubo virginianus* tibiotarsus (**c’**) shows left element due to lack of right-hand specimens. Fossil specimens (**c,d,h-k**) do not necessarily represent elements from one individual. Specimens (**a**,**b**,**e**–**g)** ©The Trustees of the Natural History Museum, London. MW: midshaft width; ML: maximum length.

**Figure 2:**
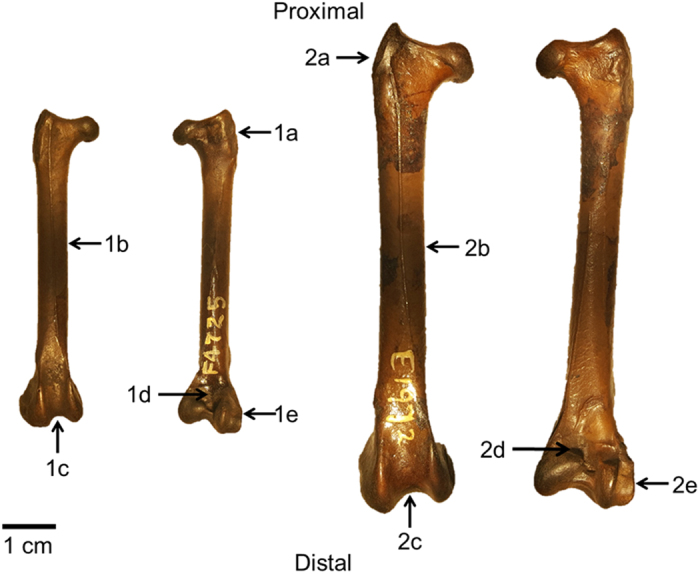
*Tyto alba* (**1a**–**e**) and *Bubo virginianus* (**2a**–**e**) femur morphology. Anterior view on left and posterior view on right for each specimen. (**1a)** Moderately developed, strong trochanter; (**1b)** Straight shaft; (**1c)** Deep, wide rotular groove; (**1d)** Shallow popliteal area; (**1e)** Strongly developed fibular condyle. (**2a)** Stout trochanter with heavily sculptured base; (**2b)** Stout, straight shaft; (**2c)** Wide, deep rotular groove; (**2d)** Deep popliteal area; (**2e)** Prominent fibular condyle.

**Figure 3:**
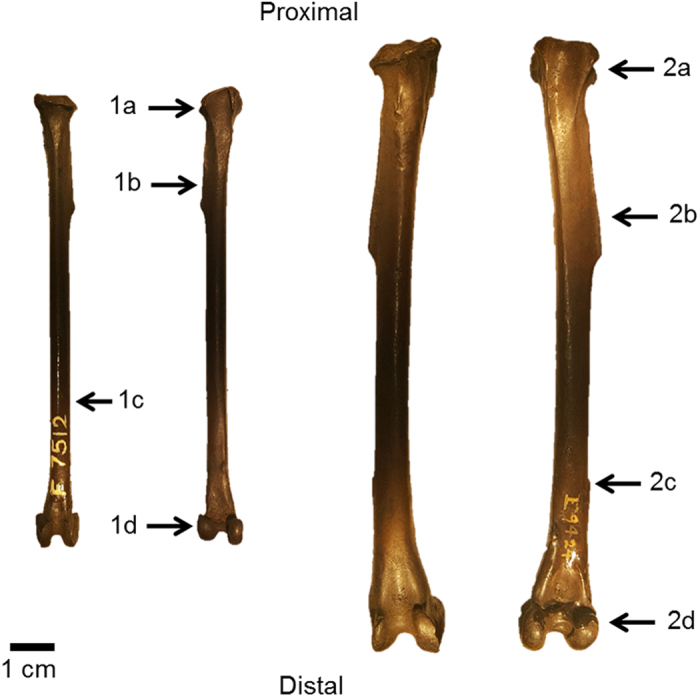
*Tyto alba* (**1a**–**d**) and *Bubo virginianus* (**2a**–**d**) tibiotarsus morphology. Anterior view on right and posterior view on left for each specimen. (**1a)** Moderately developed cnemial crests; (**1b)** Slight fibular crest; (**1c)** Fibula free of shaft; (**1d)** Distal condyles of nearly equal size. (**2a)** Moderately developed cnemial crests; (**2b)** Moderately developed fibular crest; (**2c)** Fibula fused distally to shaft; (**2d)** Distal condyles of nearly equal size.

**Figure 4:**
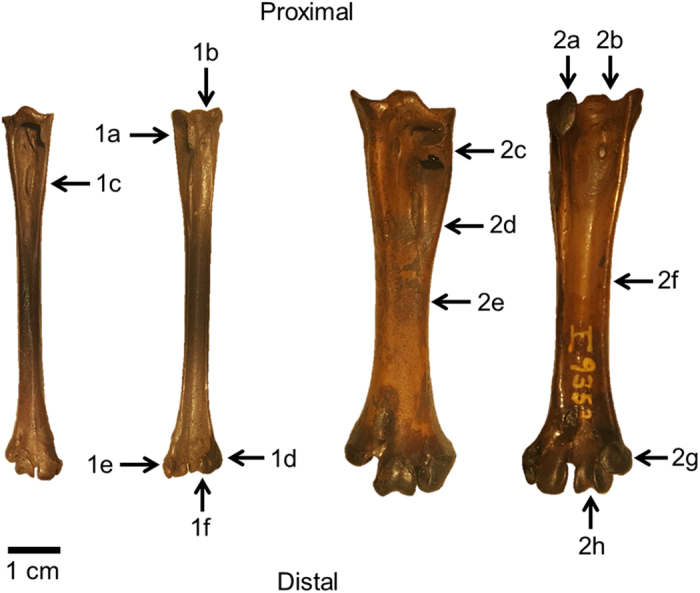
*Tyto alba* (**1a**–**f**) and *Bubo virginianus* (**2a**–**h**) tarsometatarsus morphology. Anterior view on left and posterior view on right for each specimen. (**1a)** Pronounced calcaneal ridge; (**1b)**. Hypotarsus with wide, open tendinal canal; (**1c)**. Deep anterior groove at proximal end of shaft; (**1d)**. Fourth trochlea short and deflected backward; (**1e)**. Second trochlea deflected backward; (**1f).** All trochlea closely crowded, little extended. (**2a)** Pronounced calcaneal ridge; (**2b)**. Wide, deep tendinal groove; (**2c)**. Arched, ring-like canal on anterior face of shaft at proximal end; (**2d)**. Deep anterior groove; (**2e**). Shaft wide and flat; (**2f**). Deep posterior groove; (**2g**). Fourth trochlea elevated; (**2h**). Second and third trochlea on same level.

Among the three hind limb elements, tarsometatarsi present the greatest morphological disparity across species while femora and tibiotarsi display far less variability (Fig. 1). To highlight synapomorphies of each family, morphologies of the hind limb elements of *T. alba* and *B. virginianus* are described here (Figs 2, 3 and 4).

The femur of *T. alba* has a strong, moderately developed trochanter and a straight shaft; distally, the rotular groove is deep and wide and the popliteal area is shallow; the fibular condyle is also strongly developed. The femur of *B. virginianus* presents with a stout trochanter with a heavily sculpted base and a stout, straight shaft; the rotular groove is deep and wide, as is the popliteal area; the fibular condyle is very prominent (Fig. 2).

In the tibiotarsus, *T. alba* has moderately developed cnemial crests, and a slight fibular crest; the fibula is free of the shaft and the distal condyles are of nearly equal size. In the tibiotarsus of *B. virginianus*, the cnemial and fibular crests are also moderately developed, though the fibula is fused distally to the shaft and the distal condyles are also of nearly equal size (Fig. 3).

*Tyto alba* tarsometatarsi generally present with a pronounced calcaneal ridge, a hypotarsus with a wide, open tendinal canal, a deep groove on the anterior proximal end, and closely crowded trochleae where the second and fourth trochleae are deflected backward and the fourth is shorter. The tarsometatarsus of *B. virginianus* also presents with a prominent calcaneal ridge, wide, deep tendinal grooves on the anterior and posterior, a wide, flat shaft, an arched canal on the anterior face of the shaft at the proximal end, and an elevated fourth trochlea (Fig. 4).

**Figure 5:**
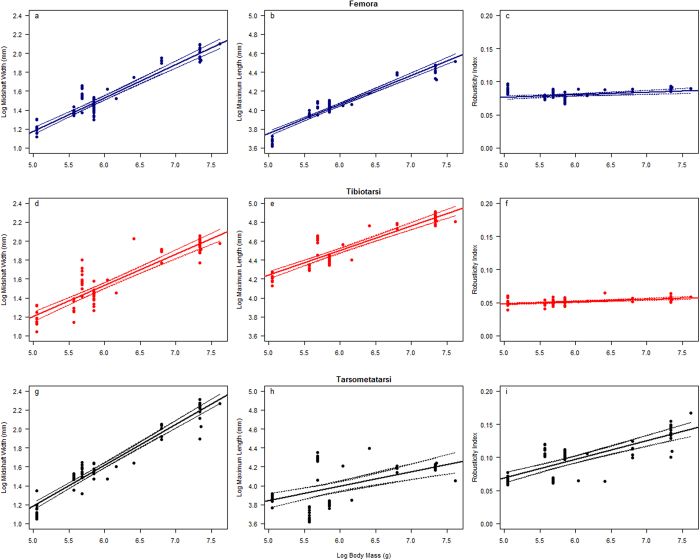
Strigiform hind limb dimensions plotted against log body mass (g). Plots show variation in midshaft width (**a**,**d**,**g**), maximum length (**b**,**e**,**h**) and robusticity index (**c**,**f**,**i**) for the femora (**a**–**c**), tibiotarsus (**d**–**f**), and tarsometatarsus (**g**–**i**). Dotted lines indicate 95% confidence intervals.

In the species studied here, midshaft width of the femur, tibiotarsus, and tarsometatarsus all demonstrate a statistically significant scaling relationship with body mass (*R*^*2*^ between 0.8228–0.9324; p < 0.0001; Fig. 5; Table 2). Midshaft width scales isometrically to body mass in femora (*a* = 0.3526 ± 0.0291, *R*^*2*^ = 0.8693) and tibiotarsi (*a* = 0.3287 ± 0.0358, *R*^*2*^ = 0.8228). Tarsometatarsi scale with positive allometry (*a* = 0.4314 ± 0.0290, *R*^*2*^ = 0.9324), becoming wider in more massive birds (Fig. 5; Table 2). The direction of allometry does not change following pgls, and all results remain significant (Table 3).

Maximum length of the femur, tibiotarsus, and tarsometatarsus all show a significant relationship with body mass (p < 0.0001; Fig. 5; Table 2). Femora scale close to isometry, however isometry can be rejected in favour of slight negative allometry (*a* = 0.3067 ± 0.0209; *R*^*2*^ = 0.9066). In contrast, tibiotarsi scale with negative allometry and closer to elastic similarity (*a* = 0.2587 ± 0.0294; *R*^*2*^ = 0.8106). Tarsometatarsi maximum lengths scale against body mass with strong negative allometry (*a* = 0.1529 ± 0.0608), indicating a much shorter length relative to body mass, although this element does display a great deal of variability across species (*R*^*2*^ = 0.2748) (Fig. 5; Table 2). Following pgls, the direction of allometry does not change in the femur or the tarsometatarsus, but while the slope (*a*) remains similar, the increased size of the confidence intervals means that isometry cannot be rejected for the tibiotarsus. All results remain significant, although the p-value for the tarsometatarsus is much higher than before, at 0.03773 (Table 3).

Robusticity indices on a semi-log plot for femora (*a* = 0.0038 ± 0.0022; *R*^*2*^ = 0.1086) and tibiotarsi (*a* = 0.0036 ± 0.0012; *R*^*2*^ = 0.3281) show weak positive allometry (p < 0.001) close to an exponent of 0, as expected for isometry. In contrast, the tarsometatarsi robusticity index scales with strong (p < 0.0001) positive allometry (*a* = 0.0279 ± 0.0059; *R*^*2*^ = 0.5778) (Fig. 5; Table 2). Note that RI *R*^*2*^ values are generally less than limb length regression *R*^*2*^ values, although tarsometatarsi RI scales to body mass with *R*^*2*^ = 0.5778 (Table 2). Following pgls, while the slopes (*a*) of the regressions remain similar for both the femur and the tibiotarsus, the increased size of the confidence intervals means that weak positive allometry cannot be rejected, although both elements scale close to isometry. Furthermore, the result for the femur is no longer significant (p = 0.05671). Allometry for the tarsometatarsus remains positive and significant (Table 3).

## Discussion

In birds, the hind limbs serve as the principal support structure for the body mass. In animals of broadly similar overall morphology but different sizes, as in the owls studied here, a proportional relationship between body mass and the dimensions of the supporting skeletal structure would seem obvious. One would expect that as the birds become larger, stress in the hind limb elements would increase proportionately and the corresponding dimensional adjustments would be evident in those elements. Previous large scale studies of bird hind limb scaling have shown that tarsometatarsus length and frequently tibiotarsus length scale against body mass with strong positive allometry, while femur length scales with isometry^21^,^22^,^23^.

In this study, owl femora maintain isometry in midshaft width and fall just short of isometry when maintaining length as body mass increases, indicating that the dimensional proportions of the femur remain almost the same relative to body size. Tibiotarsi display isometry in midshaft width and negative allometry in maximum length, close to the exponent of 0.25 expected for elastic similarity, as body mass increases. Owl tibiotarsi therefore maintain their midshaft width proportions while maximum length decreases slightly across species. Robusticity indices for both femora and tibiotarsi confirm that these two elements scale with very slight positive allometry to body mass but close to the expected exponent of 0 for isometry. The most pronounced changes in shape dimensions are seen in the owl tarsometatarsus. Here, midshaft width scales with positive allometry and maximum length scales with strong negative allometry, indicating that the tarsometatarsus becomes shorter and stouter as owl species increase in body mass. As therefore expected, robusticity index scales with strong positive allometry, meaning that as the owls studied here increase in size, their tarsometatarsi become relatively more robust. These results remain significant even after accounting for the phylogenetic similarities between species, although the large confidence intervals and the fact that Pagel’s lambda is often reported as 0 or 1 suggest that these analyses suffer from issues relating to low sample size, and future analyses would benefit from considering more species from both strigiform families.

Campbell^25^ noted the sheer variety of tarsometatarsus morphologies across the class Aves, and that different functions modify this element dramatically. He further acknowledged that birds with a similar tarsometatarsus morphotype, and subsequent functional similarity, would likely have a consistent relationship between their body mass and the dimensions of this element. We find this to indeed be the case in our clade-specific study of Strigiformes. Moreover, owls are most frequently represented in the fossil record by their durable tarsometatarsi^26^. Given the correlation between tarsometatarsus width and body mass, where midshaft width increases as owls become more massive, this measurement alone may allow researchers to estimate prehistoric owl body masses. Yet this conclusion contradicts Field^27^, who asserted that avian skeletal element dimensions may be influenced more by ecology than biomechanics, citing the short, robust owl tarsometatarsus as an example of adaptation for prey handling efficiency. Field concluded that such elements make poor body mass estimates and found instead that femur length was a stronger correlate to body mass in Strigiformes than other skeletal dimensions.

However, our results broadly tally with previous studies in demonstrating that avian hind limb elements may show adaptations that are clade-specific. For example, the femur lengths of palaeognaths (14 species, including tinamous, kiwis, moas, cassowaries, emus, and ostriches) and sphenisciformes (penguins, 7 species), scale with slight negative allometry^28^. In waterfowl and other species classified as non-running birds (20 taxonomically disparate taxa), all major hind limb elements scale with isometry^24^. Conversely, in a study of 8 taxonomically disparate “running birds”, the horizontal bones of the limbs scale closer to elastic similarity^23^. The tendency of the owl tibiotarsi to also scale close to elastic similarity suggests a role for this element in resisting deflection during locomotion and prey capture, much as the limb bones of running birds appear to be resisting functional loading in a similar manner^23^,^25^.

The tarsometatarsus plays a role in skeletal support, locomotion and hunting, and prey capture. Since Strigiformes ambush their prey close to the ground, prey may not be dead or seriously injured upon seizure, leaving open the possibility for a struggle within the bird’s talons. Furthermore, many owls must be able to restrain their squirming prey while operating under darkness. They have also been shown to strike at prey with far more force than is necessary to kill it, a behaviour which is attributed to the need to penetrate snow or leaf litter to capture hidden prey^29^, and may necessitate proportionally stronger foot bones. In a study comparing the hind limbs of equivalently sized nocturnal owls and diurnal hawks, which tend to predate on similarly-sized mammals, owls demonstrated greater grip force^30^. Sesamoids, or ossified tendons in areas of high stress, were virtually absent in hawks but well-developed in owls, spanning the length of each hind limb muscle studied. The greater force output of owl hind limbs may be a result of the presence of sesamoids in the muscles in combination with a short, robust tarsometatarsus, in contrast to the long, gracile hawk tarsometatarsus which is well adapted for high-velocity movements^30^. This greater force production may be an adaptation to prevent prey escaping an owl’s grip in the dark, whereas hawks do not require this level of force since as diurnal hunters they may visualise their prey should it escape from their talons.

## Conclusion

In the case of Strigiformes, we find a differing scaling relationship for each element of the hind limb. The tarsometatarsus shows a clear allometric scaling relationship, becoming relatively stouter, shorter and more robust as species body size increases. In contrast, the femur and tibiotarsus show only a slight increase in robusticity as size increases, facilitated by slight negative allometry in length, particularly in the tibiotarsus, whilst midshaft width maintains geometric similarity. These results suggest that the tarsometatarsus plays a primary role in withstanding functional loading in owls. Tarsometatarsus robusticity (*R*^*2*^ = 0.5778) scales most closely with body mass and its width is also the best indicator of body mass based on dimensional measurements alone (*R*^*2*^ = 0.9324). Therefore, using measurements from tarsometatarsi, the element most commonly recovered from the fossil record of owls, body mass estimates of extinct taxa may be possible.

## Materials and Methods

The collections of the George C. Page Museum at the La Brea Tar Pits in Los Angeles, California, and the Natural History Museum at Tring, Hertfordshire were utilized in order to measure dimensions and evaluate osteological features among owl species representing both the Strigidae and the Tytonidae. Exceptionally preserved Pleistocene subfossil material from the Page Museum, where at least nine species of owls are represented by over 7,500 specimens in the collections, provided an opportunity to gauge morphological variation within species.

Limb bones from six species were measured from the Rancho La Brea fossil assemblage at the Page Museum: *Asio flammeus* (short-eared owl), *Asio otus* (long-eared owl), *Athene cunicularia* (burrowing owl), *Bubo virginianus* (great horned owl), *Tyto alba* (barn owl), and the extinct *Oraristrix brea* (La Brea owl). A total of seventy-nine femora, sixty-three tibiotarsi, and fifty-five tarsometatarsi were evaluated (Table 1 and Supplementary Table S1).

From the collections in Tring, data for a single specimen representing each of ten species were acquired of the following species: *As. flammeus, As. otus, At. cunicularia, B. virginianus, Ketupa ketupu* (Malay fish owl), *Nyctea scandiaca* (snowy owl) and *Strix aluco* (tawny owl) representing the Strigidae; and *T. alba, T. capensis* (African grass owl), and *T. novaehollandiae* (Australian masked owl) representing the Tytonidae (Supplementary Table S2).

Unbroken adult specimens of the three major hind limb elements: femur, tibiotarsus, and tarsometatarsus were measured in millimetres with digital callipers. Digital photographs of each specimen were obtained. Right limb bones were utilized, where possible, in order to ensure each element belonged to an individual specimen. Maximum length and midshaft width were determined from each element (Fig. 1; Supplementary Tables S1 and S2).

The tarsometatarsi maximum length is defined as the distance between the calcaneal ridge and the tip of the trochlea (Fig. 1a). In tibiotarsi, maximum length is defined as the distance between the cnemial crests and the distal condyle (Fig. 1a’). Femoral maximum length was defined as the distance between the tip of the trochanter and the end of the distal condyle. In all, midshaft width was defined as the mediolateral width at half the total length (Fig. 1a”).

In order to assess scaling patterns among hind limb elements, the robusticity of each element was obtained by calculating a robusticity index: the ratio of midshaft width to maximum length (Supplementary Tables S1 and S2). The values acquired for robusticity were plotted against body mass (taken as the average of male and female body masses; body mass for *Tyto alba* is of *Tyto alba guttata* subspecies) in grams using values obtained from Dunning^31^, and Campbell and Bochenski^32^ for *Oraristrix brea* (which they calculated using the formula for ordinary least squares regression: log(y) = 2.548·log(x) − 0.414, where y = mass in grams and x = least shaft circumference of femur in mm; formula taken from the results of a study of the relationship of hindlimb bone dimensions to weight in birds by Campbell & Marcus^25^), in R (v. 3.2.4)^33^ on a semi-log plot, whereas midshaft width and maximum length were plotted in the same manner on log/log plots. Ordinary least squares (OLS) regression analyses were performed on the data to display the strength of the correlation (*R*^*2*^) between the variables and the statistical significance of those correlations (p-value). Ninety-five percent confidence intervals were calculated for each regression and the scaling exponent, intercept, *R*^*2*^ value, and p-value were tabulated (Fig. 5; Table 2). To ensure that the results obtained were not merely due to the close phylogenetic relatedness of the species studies, the regressions were repeated using phylogenetic generalised least squares (pgls; Table 3) using the R package caper^34^. A maximum clade credibility tree was created in TreeAnnotator package in Beast 2.1.2 (ref. 35) from a set of 1,000 trees downloaded from birdtree.org^8^. Pagel’s lambda (λ) varies between 0 and 1, and indicates the level of phylogenetic signal in the data, where 0 represents no phylogenetic signal.

## Additional Information

**How to cite this article**: Madan, M. A. *et al*. Scaling and functional morphology in strigiform hind limbs. *Sci. Rep.* **7**, 44920; doi: 10.1038/srep44920 (2017).

**Publisher's note:** Springer Nature remains neutral with regard to jurisdictional claims in published maps and institutional affiliations.

## Supplementary Material

Supplementary Information

## Figures and Tables

**Table 1:** Page Museum specimens.

Species	Number of Specimens		
	Femora	Tibiotarsi	Tarsometatarsi
*Asio flammeus*	40	20	10
*Bubo virginianus*	10	14	10
*Tyto alba*	10	10	10
*Athene cunicularia*	10	10	10
*Asio otus*	5	5	10
*Oraristrix brea*	4	4	5
TOTAL	79	63	55

**Table 2:** Results of ordinary least squares regressions of bone dimensions to log body mass.

	Slope (*a*)	Int (*b*)	Lower CI	Upper CI	Allometry	p	Adjusted *R*^*2*^
**Midshaft Width**							
Femora	0.35264	−0.5858	0.32352	0.38176	Isometry	<2.2e-16	0.8693
Tibiotarsus	0.32873	−0.4411	0.29283	0.36463	Isometry	<2.2e-16	0.8228
Tarsometatarsus	0.43135	−0.97061	0.40233	0.46037	Strong Positive	<2.2e-16	0.9324
**Maximum Length**							
Femora	0.30665	2.21889	0.28569	0.32761	Negative	<2.2e-16	0.9066
Tibiotarsus	0.25872	2.94832	0.2293	0.28814	Negative	<2.2e-16	0.8106
Tarsometatarsus	0.15293	3.0768	0.09207	0.21379	Strong Negative	4.42E-06	0.2748
**Robusticity Index**							
Femora	0.003772	0.057464	0.001568	0.005976	Isometry	0.0009433	0.1086
Tibiotarsus	0.003611	0.029908	0.0024098	0.0048122	Isometry	7.15E-08	0.3281
Tarsometatarsus	0.027883	−0.070185	0.021959	0.033807	Positive	1.26E-13	0.5778

Equations of the form *y* = *bx*^*a*^. CI, confidence interval = 2 × std. error.

**Table 3:** Results of phylogenetic generalised least squares regressions of bone dimensions to log body mass.

	Slope (*a*)	Int (*b*)	Lower CI	Upper CI	Allometry	p	Adjusted *R*^*2*^	λ
**Midshaft Width**								
Femora	0.333701	−0.43885	0.280587	0.386815	Isometry	0.000001508	0.9457	1
Tibiotarsus	0.346036	−0.506137	0.235684	0.456388	Isometry	0.0002401	0.8088	0.656
Tarsometatarsus	0.401652	−0.840073	0.33323	0.470074	Strong Positive	0.000002532	0.9383	0
**Maximum Length**								
Femora	0.277359	2.37367	0.225013	0.329705	Negative	0.000005497	0.9252	0
Tibiotarsus	0.273012	2.869966	0.188142	0.357882	Isometry	0.0002018	0.8178	0.791
Tarsometatarsus	0.173424	3.02773	0.033932	0.312916	Strong Negative	0.03773	0.3654	0.909
**Robusticity Index**								
Femora	0.004655	0.056677	0.0004713	0.0088389	Isometry	**0.05671**	0.3051	1
Tibiotarsus	0.003823	0.0286026	0.0028271	0.0048191	Isometry	0.00005871	0.8655	0
Tarsometatarsus	0.024239	−0.059415	0.0058656	0.0426132	Positive	0.02978	0.3985	0.753

Equations of the form *y* = *bx*^*a*^. CI, confidence interval = 2 × std. error. Bold values indicate results that are not significant (p > 0.05). λ = Pagel’s lambda.
