# Supplementary material for: Scaling and functional morphology in strigiform hind limbs
Source: Sci Rep. 2017 Mar 22;7:44920. doi: 10.1038/srep44920 (PMC5361165; doi:10.1038/srep44920)
Supplement: Supplementary Information [file srep44920-s1.docx]

**SCALING AND FUNCTIONAL MORPHOLOGY IN STRIGIFORM HIND LIMBS**

Meena A. Madan^1*^, Emily J. Rayfield^1^, and Jen A. Bright^2,3^

^1^School of Earth Sciences, University of Bristol, Bristol, United Kingdom

^2^School of Geosciences, University of South Florida, Tampa, Florida, USA

^3^Center for Virtualization and Applied Spatial Technologies, University of South Florida, Tampa, Florida, USA

*Corresponding Author: mm13970@my.bristol.ac.uk

**Supplementary Information**

*Supplementary Table S1. Page Museum specimens*

| FEMORA | | | | | | Total: 79 |
| --- | --- | --- | --- | --- | --- | --- |
| Taxon | **Body Mass**  **(g)** | **Catalogue Number** | **Midshaft Width (mm)** | **Maximum Length (mm)** | **Robusticity Index** | |
| *Asio flammeus* | 346.5 | F5335 | 4.41 | 57.11 | 0.0772 | |
|  |  | F5053 | 4.05 | 57.09 | 0.0709 | |
|  |  | E9245 | 4.02 | 56.12 | 0.0716 | |
|  |  | F5034 | 4.11 | 56.42 | 0.0728 | |
|  |  | F5332 | 4.29 | 55.10 | 0.0779 | |
|  |  | E9239 | 4.13 | 56.26 | 0.0734 | |
|  |  | E8926 | 4.18 | 57.68 | 0.0725 | |
|  |  | F5326 | 3.96 | 55.01 | 0.0720 | |
|  |  | F5018 | 4.51 | 60.40 | 0.0747 | |
|  |  | F5132 | 4.11 | 53.47 | 0.0769 | |
|  |  | F5031 | 4.58 | 56.94 | 0.0804 | |
|  |  | E9325 | 4.15 | 53.97 | 0.0769 | |
|  |  | E8879 | 4.15 | 57.76 | 0.0718 | |
|  |  | F1916 | 3.98 | 58.25 | 0.0683 | |
|  |  | E8773 | 4.34 | 57.81 | 0.0751 | |
|  |  | E8780 | 3.96 | 55.34 | 0.0716 | |
|  |  | F5129 | 4.38 | 56.26 | 0.0779 | |
|  |  | F5021 | 4.04 | 58.37 | 0.0692 | |
|  |  | E9311 | 4.20 | 57.27 | 0.0733 | |
|  |  | E9320 | 3.95 | 53.48 | 0.0739 | |
|  |  | F5054 | 4.23 | 54.37 | 0.0778 | |
|  |  | F5041 | 4.62 | 54.90 | 0.0842 | |
|  |  | F5025 | 4.08 | 54.93 | 0.0743 | |
|  |  | F5063 | 3.78 | 55.13 | 0.0686 | |
|  |  | F5123 | 3.66 | 55.15 | 0.0664 | |
|  |  | F5056 | 3.79 | 55.20 | 0.0687 | |
|  |  | F5043 | 4.25 | 56.28 | 0.0755 | |
|  |  | E9056 | 4.16 | 54.97 | 0.0757 | |
|  |  | K2912 | 4.28 | 55.92 | 0.0765 | |
|  |  | F5116 | 4.00 | 54.27 | 0.0737 | |
|  |  | F5343 | 4.63 | 59.54 | 0.0778 | |
|  |  | F5127 | 4.19 | 54.47 | 0.0769 | |
|  |  | F5333 | 4.34 | 55.60 | 0.0781 | |
|  |  | F5052 | 3.96 | 57.07 | 0.0694 | |
|  |  | E8809 | 4.19 | 57.17 | 0.0733 | |
|  |  | F5024 | 3.95 | 54.86 | 0.0720 | |
|  |  | E3544 | 3.95 | 55.60 | 0.0710 | |
|  |  | F5499 | 3.82 | 55.25 | 0.0691 | |
|  |  | F5027 | 4.06 | 56.95 | 0.0713 | |
|  |  | F5253 | 4.11 | 53.30 | 0.0771 | |
| *Bubo virginianus* | 1543 | E9662 | 7.47 | 82.68 | 0.0903 | |
|  |  | E9639 | 6.99 | 82.17 | 0.0851 | |
|  |  | E9797 | 6.93 | 81.30 | 0.0852 | |
|  |  | E9627 | 7.11 | 82.52 | 0.0862 | |
|  |  | E9611 | 7.84 | 84.61 | 0.0927 | |
|  |  | F4875 | 7.48 | 82.19 | 0.0910 | |
|  |  | F4868 | 7.77 | 87.26 | 0.0890 | |
|  |  | E9421 | 8.07 | 87.11 | 0.0926 | |
|  |  | E1537 | 7.66 | 88.17 | 0.0869 | |
|  |  | E1972 | 8.12 | 86.85 | 0.0935 | |
| *Tyto alba* | 294 | E9161 | 4.80 | 58.52 | 0.0820 | |
|  |  | E9085 | 5.10 | 58.66 | 0.0869 | |
|  |  | E8987 | 4.73 | 56.35 | 0.0839 | |
|  |  | F4846 | 4.64 | 58.80 | 0.0789 | |
|  |  | F4750 | 4.64 | 56.52 | 0.0821 | |
|  |  | F4655 | 5.12 | 59.01 | 0.0868 | |
|  |  | K2916 | 4.58 | 55.97 | 0.0818 | |
|  |  | F1615 | 5.24 | 59.90 | 0.0875 | |
|  |  | F4725 | 5.23 | 58.77 | 0.0890 | |
|  |  | F4741 | 5.18 | 58.76 | 0.0882 | |
| *Athene cunicularia* | 155 | K9343 | 3.67 | 38.09 | 0.0964 | |
|  |  | F5281 | 3.40 | 38.30 | 0.0888 | |
|  |  | F5483 | 3.17 | 37.16 | 0.0853 | |
|  |  | E9127 | 3.68 | 39.12 | 0.0941 | |
|  |  | F5284 | 3.32 | 41.34 | 0.0803 | |
|  |  | F5851 | 3.28 | 39.51 | 0.0830 | |
|  |  | F5874 | 3.39 | 37.62 | 0.0901 | |
|  |  | F5282 | 3.06 | 37.56 | 0.0815 | |
|  |  | F5477 | 3.37 | 40.22 | 0.0838 | |
|  |  | F5838 | 3.29 | 39.35 | 0.0836 | |
| *Asio otus* | 262 | F5423 | 3.82 | 52.67 | 0.0725 | |
|  |  | K9359 | 3.90 | 51.34 | 0.0760 | |
|  |  | F5124 | 4.20 | 54.32 | 0.0773 | |
|  |  | E9691 | 3.86 | 51.90 | 0.0744 | |
|  |  | F5109 | 3.96 | 51.35 | 0.0771 | |
| *Oraristrix brea* | 900 | E9647 | 6.88 | 80.84 | 0.0851 | |
|  |  | E9909 | 6.83 | 80.14 | 0.0852 | |
|  |  | E9439 | 6.66 | 79.07 | 0.0842 | |
|  |  | B9889 | 7.05 | 79.34 | 0.0889 | |

| TIBIOTARSI | | | | | | Total: 63 |
| --- | --- | --- | --- | --- | --- | --- |
| **Taxon** | **Body Mass (g)** | **Catalogue Number** | **Midshaft Width (mm)** | **Maximum Length (mm)** | **Robusticity Index** | |
| *Asio flammeus* | 346.5 | F7657 | 4.12 | 80.18 | 0.0514 | |
|  |  | F7644 | 4.30 | 83.63 | 0.0514 | |
|  |  | F7495 | 4.84 | 83.84 | 0.0577 | |
|  |  | F7724 | 3.81 | 82.16 | 0.0464 | |
|  |  | F7639 | 4.00 | 81.23 | 0.0492 | |
|  |  | E8729 | 4.23 | 77.18 | 0.0548 | |
|  |  | F7640 | 4.01 | 79.24 | 0.0506 | |
|  |  | F7602 | 4.40 | 84.61 | 0.0520 | |
|  |  | F7631 | 3.55 | 79.64 | 0.0446 | |
|  |  | F7655 | 3.71 | 83.52 | 0.0444 | |
|  |  | F7736 | 4.31 | 83.79 | 0.0514 | |
|  |  | F7718 | 4.18 | 77.89 | 0.0537 | |
|  |  | F7663 | 4.32 | 83.56 | 0.0517 | |
|  |  | F7610 | 3.99 | 79.75 | 0.0500 | |
|  |  | F7588 | 4.15 | 82.70 | 0.0502 | |
|  |  | F7734 | 4.16 | 82.80 | 0.0502 | |
|  |  | E8697 | 4.13 | 80.80 | 0.0511 | |
|  |  | F7695 | 4.23 | 79.79 | 0.0530 | |
|  |  | F7583 | 4.31 | 83.38 | 0.0517 | |
|  |  | F7702 | 4.37 | 86.51 | 0.0505 | |
| *Bubo virginianus* | 1543 | E9970 | 6.54 | 119.20 | 0.0549 | |
|  |  | E9560 | 7.20 | 127.26 | 0.0566 | |
|  |  | K9533 | 7.43 | 127.31 | 0.0584 | |
|  |  | E9434 | 7.49 | 125.35 | 0.0598 | |
|  |  | K9515/K9518 | 7.81 | 122.84 | 0.0636 | |
|  |  | K3127 | 7.07 | 129.52 | 0.0546 | |
|  |  | F7437 | 6.65 | 119.30 | 0.0557 | |
|  |  | F7433 | 7.63 | 132.60 | 0.0575 | |
|  |  | F28 | 7.08 | 123.75 | 0.0572 | |
|  |  | F27 | 6.95 | 124.07 | 0.0560 | |
|  |  | E9893 | 6.99 | 135.31 | 0.0517 | |
|  |  | E9568 | 7.30 | 132.04 | 0.0553 | |
|  |  | E9424 | 7.58 | 135.71 | 0.0559 | |
|  |  | E9349 | 7.17 | 126.79 | 0.0566 | |
| *Tyto alba* | 294 | E1671 | 5.55 | 100.20 | 0.0554 | |
|  |  | E9445 | 4.48 | 101.98 | 0.0439 | |
|  |  | F7520 | 4.89 | 104.88 | 0.0466 | |
|  |  | F7512 | 4.68 | 105.07 | 0.0445 | |
|  |  | F7526 | 6.06 | 103.72 | 0.0584 | |
|  |  | F6424 | 5.18 | 104.42 | 0.0496 | |
|  |  | E9140 | 4.85 | 100.00 | 0.0485 | |
|  |  | F6420 | 4.89 | 97.54 | 0.0501 | |
|  |  | E8719 | 4.76 | 101.07 | 0.0471 | |
|  |  | F8138 | 5.37 | 103.03 | 0.0521 | |
| *Athene cunicularia* | 155 | F8019 | 2.84 | 72.12 | 0.0394 | |
|  |  | F8048 | 3.75 | 65.22 | 0.0575 | |
|  |  | F8030 | 3.28 | 65.61 | 0.0500 | |
|  |  | F8064 | 3.49 | 67.21 | 0.0519 | |
|  |  | F8192 | 3.27 | 64.75 | 0.0505 | |
|  |  | F7799 | 3.08 | 65.59 | 0.0470 | |
|  |  | F8221 | 3.09 | 67.34 | 0.0459 | |
|  |  | F8233 | 3.15 | 68.39 | 0.0461 | |
|  |  | F8182 | 3.49 | 71.00 | 0.0492 | |
|  |  | F7658 | 3.25 | 71.11 | 0.0457 | |
| *Asio otus* | 262 | F7719 | 3.64 | 76.47 | 0.0476 | |
|  |  | F7680 | 3.50 | 74.41 | 0.0470 | |
|  |  | E9005 | 3.55 | 77.49 | 0.0458 | |
|  |  | F7691 | 3.91 | 72.83 | 0.0537 | |
|  |  | F7673 | 3.14 | 77.25 | 0.0406 | |
| *Oraristrix brea* | 900 | E9932 | 6.79 | 120.10 | 0.0565 | |
|  |  | E9888 | 6.63 | 118.82 | 0.0558 | |
|  |  | E9414 | 6.68 | 119.26 | 0.0560 | |
|  |  | E9267 | 5.86 | 113.19 | 0.0518 | |

| TARSOMETATARSI | | | | | | Total:  55 |
| --- | --- | --- | --- | --- | --- | --- |
| **Taxon** | **Body Mass**  **(g)** | **Catalogue Number** | **Midshaft Width (mm)** | **Maximum Length (mm)** | **Robusticity Index** | |
| *Asio flammeus* | 346.5 | G4729 | 4.61 | 44.58 | 0.1034 | |
|  |  | K1049 | 5.15 | 46.22 | 0.1114 | |
|  |  | G4822 | 4.60 | 44.30 | 0.1038 | |
|  |  | G4718 | 4.61 | 46.56 | 0.0990 | |
|  |  | G4699 | 4.71 | 44.18 | 0.1066 | |
|  |  | G4853 | 4.85 | 45.53 | 0.1065 | |
|  |  | E8730 | 5.11 | 46.51 | 0.1099 | |
|  |  | G4720 | 4.35 | 45.10 | 0.0965 | |
|  |  | G4870 | 4.70 | 46.72 | 0.1006 | |
|  |  | G4854 | 4.66 | 45.23 | 0.1030 | |
| *Bubo virginianus* | 1543 | E9426 | 9.66 | 65.83 | 0.1467 | |
|  |  | E9668 | 8.29 | 64.38 | 0.1288 | |
|  |  | E9652 | 9.55 | 66.18 | 0.1443 | |
|  |  | E9907 | 9.28 | 65.92 | 0.1408 | |
|  |  | E9989 | 9.38 | 68.52 | 0.1369 | |
|  |  | E9665 | 9.72 | 65.05 | 0.1494 | |
|  |  | E9926 | 10.07 | 65.07 | 0.1548 | |
|  |  | E9353 | 9.22 | 67.87 | 0.1358 | |
|  |  | E9637 | 8.85 | 66.28 | 0.1335 | |
|  |  | E9787 | 9.45 | 64.93 | 0.1455 | |
| *Tyto alba* | 294 | G4199 | 5.19 | 77.52 | 0.0670 | |
|  |  | G4217 | 5.06 | 73.01 | 0.0693 | |
|  |  | G4251 | 4.57 | 74.69 | 0.0612 | |
|  |  | G4223 | 4.67 | 71.50 | 0.0653 | |
|  |  | G4196 | 4.41 | 72.14 | 0.0611 | |
|  |  | G4244 | 4.58 | 74.84 | 0.0612 | |
|  |  | G4268 | 4.94 | 72.09 | 0.0685 | |
|  |  | G4231 | 4.69 | 73.37 | 0.0639 | |
|  |  | G4215 | 4.83 | 71.97 | 0.0671 | |
|  |  | G4257 | 4.46 | 70.73 | 0.0631 | |
| *Athene cunicularia* | 155 | G4462 | 3.32 | 46.49 | 0.0714 | |
|  |  | G4581 | 3.29 | 49.74 | 0.0661 | |
|  |  | G4668 | 3.18 | 47.20 | 0.0674 | |
|  |  | K1444 | 3.19 | 50.11 | 0.0637 | |
|  |  | E9513 | 2.90 | 49.53 | 0.0586 | |
|  |  | E9036 | 2.96 | 48.72 | 0.0608 | |
|  |  | G4552 | 3.01 | 48.45 | 0.0621 | |
|  |  | E9496 | 3.06 | 47.43 | 0.0645 | |
|  |  | G4579 | 3.84 | 49.69 | 0.0773 | |
|  |  | G4639 | 2.86 | 46.28 | 0.0618 | |
| *Asio otus* | 262 | K9275 | 4.43 | 38.94 | 0.1138 | |
|  |  | K9274 | 4.45 | 43.80 | 0.1016 | |
|  |  | K9297 | 4.62 | 38.98 | 0.1185 | |
|  |  | K5272 | 4.51 | 43.82 | 0.1029 | |
|  |  | K9272 | 4.62 | 41.17 | 0.1122 | |
|  |  | G4836 | 4.44 | 43.19 | 0.1028 | |
|  |  | G4846 | 4.34 | 38.54 | 0.1126 | |
|  |  | G4811 | 3.86 | 37.91 | 0.1018 | |
|  |  | G4864 | 4.46 | 42.28 | 0.1055 | |
|  |  | G4810 | 4.42 | 39.86 | 0.1109 | |
| *Oraristrix brea* | 900 | K9621 | 7.78 | 62.72 | 0.1240 | |
|  |  | G3933 | 7.68 | 67.39 | 0.1140 | |
|  |  | E9416 | 7.44 | 65.94 | 0.1128 | |
|  |  | E9575 | 6.84 | 65.70 | 0.1041 | |
|  |  | G3958 | 6.63 | 66.63 | 0.0995 | |

*Supplementary Table S2. Tring Museum specimens*

| Taxon  (Catalogue Number) | Body Mass  (g) | Element | Midshaft  Width  (mm) | Maximum  Length  (mm) | Robusticity Index |
| --- | --- | --- | --- | --- | --- |
| *Tyto novaehollandiae*  (S/1954-30-84) | 609 | Femur | 5.72 | 65.36 | 0.0875 |
|  |  | Tibiotarsus (+ fibula) | 7.59 | 116.7 | 0.0650 |
|  |  | Tarsometatarsus | 5.16 | 81.27 | 0.0635 |
| *Tyto alba*  (S/1986-36-8) | 294 | Femur | 3.95 | 51.71 | 0.0764 |
|  |  | Tibiotarsus (+ fibula) | 4.12 | 85.70 | 0.0481 |
|  |  | Tarsometatarsus | 3.74 | 58.00 | 0.0645 |
| *Tyto capensis*  (1866-7-3-11) | 419 | Femur | 5.05 | 57.10 | 0.0884 |
|  |  | Tibiotarsus (+ fibula) | 4.91 | 95.64 | 0.0513 |
|  |  | Tarsometatarsus | 4.36 | 67.47 | 0.0646 |
| *Nyctea scandiaca*  (S/2012-10-1) | 2042.5 | Femur | 8.16 | 91.00 | 0.0897 |
|  |  | Tibiotarsus (+ fibula) | 7.22 | 122.5 | 0.0589 |
|  |  | Tarsometatarsus | 9.64 | 57.69 | 0.1671 |
| *Bubo virginianus*  (1850-11-14-8) | 1543 | Femur | 6.73 | 76.36 | 0.0881 |
|  |  | Tibiotarsus (+ fibula) | 5.88 | 117.1 | 0.0502 |
|  |  | Tarsometatarsus | 6.65 | 66.04 | 0.1007 |
| *Ketupa ketupu*  (1850-8-15-20) | 1564 | Femur | 6.86 | 75.10 | 0.0913 |
|  |  | Tibiotarsus (+ fibula) | 6.79 | 122.6 | 0.0554 |
|  |  | Tarsometatarsus | 7.57 | 69.54 | 0.1089 |
| *Strix aluco*  (S/1992-56-1) | 475 | Femur | 4.59 | 58.10 | 0.0790 |
|  |  | Tibiotarsus (+ fibula) | 4.28 | 81.65 | 0.0524 |
|  |  | Tarsometatarsus | 4.96 | 46.88 | 0.1058 |
| *Athene cunicularia*  (S/2014-14-2) | 155 | Femur | 3.30 | 37.90 | 0.0871 |
|  |  | Tibiotarsus (+ fibula) | 3.72 | 61.97 | 0.0600 |
|  |  | Tarsometatarsus | 2.95 | 43.23 | 0.0682 |
| *Asio flammeus*  (1930-3-24-337) | 346.5 | Femur | 3.97 | 54.06 | 0.0734 |
|  |  | Tibiotarsus (+ fibula) | 3.97 | 77.46 | 0.0513 |
|  |  | Tarsometatarsus | 4.63 | 42.91 | 0.1079 |
| *Asio otus*  (1930-3-24-331) | 262 | Femur | 4.05 | 50.72 | 0.0799 |
|  |  | Tibiotarsus (+ fibula) | 4.03 | 74.71 | 0.0539 |
|  |  | Tarsometatarsus | 4.44 | 37.16 | 0.1195 |
